# Diabetes from Nerve Strain

**Published:** 1900-06-16

**Authors:** 


### DIABETES FROM NERVE STRAIN.

Dr. Robert Saundby says that from comparisons he has made he has come to the conclusion that the mortality from diabetes is nearly twice as great among engine drivers as in the general population. The figures are as follows: Among the general population between the ages of 30 and 60 years the mortality from diabetes was 80 per 10,000 deaths, while among engine drivers of the same age (according to returns supplied by Mr. Herbert Page in regard to the London and North-Western Railway Company for a number of years) the mortality from this cause was at the rate of 130 per 10,000 deaths. This certainly seems to favour the idea that prolonged nervous strain is an important factor in the production of diabetes, an idea strongly held by most physicians who have had much to do with this disease.

